# Polymer-based nanosystems and their applications in bone anticancer therapy

**DOI:** 10.3389/fchem.2023.1218511

**Published:** 2023-07-06

**Authors:** Wanis Nafo

**Affiliations:** School of Mathematical and Statistical Sciences, University of Galway, Galway, Ireland

**Keywords:** osteosarcoma, targeted drug delivery, nanoparticles, internal stimulation, external stimulation

## Abstract

The mortality rate of bone cancer has witnessed a substantial reduction in recent years, all thanks to the advent of advanced cancer treatment modalities such as surgical intervention, radiation, and chemotherapy. Nevertheless, these popular modalities come with a set of clinical challenges, including non-specificity, side effects, and drug intolerance. In recent years, polymer-based nanosystems have emerged as a promising solution in bone anti-cancer therapy by virtue of their unique physical and chemical properties. These nanosystems can be tailored for use in different drug release mechanisms for therapeutic implementations. This review delves into the efficacy of these therapy applications in bone cancer (with a focus on one of the most common types of cancers, Osteosarcoma) treatment and their correlation with the properties of polymer-based nanosystems, in addition to their interaction with the tumor microenvironment and the biological milieu.

## 1 Introduction

Bone tumors are growths that arise in bone tissue and can be categorized as primary or metastatic. Primary tumors, such as osteosarcoma, are malignant growths that develop in the bone and are prevalent in children and teenagers. Osteosarcoma is an especially hazardous form of bone cancer, ranking as the second leading cause of tumor-related death in adolescents (Liao et al., 2021). Early detection of the disease is challenging as the symptoms may not become apparent until the tumor has advanced and caused significant pain or spontaneous fractures. The tumor’s rapid growth rate can result in substantial bone damage and limit movement, and if left unchecked, the cancer can spread to the lungs, adding to the risk and complexity of the disease (Chen et al., 2018).

Metastatic bone tumors are neoplasms that initially form in other parts of the body and subsequently spread to the bone tissue. Bone metastasis is a common occurrence, and certain types of cancer, such as breast, prostate, colon, and lung cancer, have a high propensity to metastasize to the bone (Lambert et al., 2017; Lv and Guo, 2020). Although there are some similarities between osteosarcoma and bone metastasis, the latter usually develops in the advanced stages of the tumor (Liao et al., 2021). Metastatic bone tumors and osteosarcoma share comparable tumor niches and microenvironments that arise from tumor-induced bone defects. Consequently, there is an urgent need for innovative and effective therapeutic approaches to tackle the difficulties associated with bone tumors.

The use of polymer-based nanocarriers for targeted drug delivery in orthopedic oncology applications has become increasingly popular over the last few years due to several advantages they offer. These include their physicochemical properties such as nontoxicity, water solubility, and biodegradability. Moreover, these nanocarriers have a prolonged circulation time in the bloodstream, reducing the risk of biological clearance. Additionally, they can accumulate and remain in tumor locations, and when decorated with receptor-mediated ligands, these carriers can selectively target cancer cells, further enhancing their efficacy as anti-cancer agents (Zhang et al., 2018).

The focus of this review is on the use of polymeric nanoparticles in different therapeutic applications for bone cancer, specifically, osteosarcoma. The utilization of polymer-based nanoparticles in therapeutic applications for osteosarcoma has evolved over time, offering promising advancements in the treatment of this aggressive bone cancer (Barani et al., 2021). In recent years, researchers have harnessed the unique properties of polymer nanoparticles to address several challenges in osteosarcoma therapy. These nanoparticles, composed of biocompatible polymers, can be engineered to encapsulate anticancer drugs, enhancing their delivery and specificity to tumor cells while minimizing systemic toxicity (Gui et al., 2019). Additionally, polymer-based nanoparticles have demonstrated excellent stability, prolonged circulation time, and enhanced cellular uptake, thereby maximizing therapeutic efficacy (Li, 2020; Ma and Shi, 2021). Furthermore, their versatile nature allows for the incorporation of targeting ligands and imaging agents, enabling accurate tumor detection and personalized treatment strategies (Zhou et al., 2021). Although further research is warranted, the history of using polymer-based nanoparticles in osteosarcoma therapy underscores their potential as a promising approach to improve patient outcomes and revolutionize cancer treatment paradigms.

These nanoparticles can be processed and manipulated for use in passive and/or active strategies of treatment, such as photothermal and hyperthermia therapies, pH-triggered drug delivery, and other methodologies. This review will discuss the efficacy of these therapy applications in relation to the unique properties of polymer-based nanosystems. Furthermore, the therapeutic application of these nanosystems is closely linked to their physicochemical properties, which are dependent on the synthesis methods, such as physical and chemical crosslinking, and emulsification. This review will cover different synthesis methods of polymer nanosystems and their applications in orthopedic anti-cancer therapy. Finally, this review will discuss the overall toxicity, biodistribution patterns, and functionalization of polymeric nanoparticles, as these properties are crucial in anti-cancer therapy and diagnostics.

## 2 Synthesis of polymer-based nanoparticles

Various biodegradable polymers are employed in the development of nanoparticles for drug delivery purposes in bone cancer therapy. These polymers can be categorized into two groups: synthetic, such as poly (ethylene glycol) (PEG), poly (glycolic acid) (PGA), poly (lactic acid) (PLA), polyvinyl alcohol (PVA), and poly (lactide-co-glycolic acid) (PLGA); and natural, including chitosan and gelatin. Synthetic polymers have been extensively used in medical applications such as wound sealing, sutures, and implants. Natural polymers, on the other hand, are biocompatible and have fewer cytotoxic effects due to their derivation from natural sources and proteins. In general, the current approaches to synthesize polymeric nanoparticles follow top-down or bottom-up protocols; the first revolves around the breaking of large material into a targeted size suitable for drug delivery, while the latter involves the assembly of smaller building blocks to create larger particles (Lai et al., 2014).

### 2.1 Water-in-oil- emulsification

This approach is a bottom-up strategy in which a polymer aqueous solution is added to a solvent, and then homogenized to produce the emulsion. Subsequently, this process is then followed by crosslinking—Chemical by adding a crosslinking agent (e.g., Glutaraldehyde) (Phromsopha and Baimark, 2014), or physical (cycles of freezing and thawing) (Waresindo et al., 2023). The drug loading in this approach occurs during the mixing process, in which the drug is added to the polymer aqueous solution before the homogenization stage. Furthermore, to monitor the cellular uptake by confocal microscopy, fluorescent dyes can also be added before the crosslinking process (Reisch and Klymchenko, 2016).

### 2.2 Ionotropic gelation method

Ionotropic gelation is a top-down method used to prepare nanoparticles by aggregating the polymer with multivalent counter ions, such as sodium tripolyphosphate (TPP). The procedure begins with dissolving the used polymer in a dilute acidic solution, followed by the addition of TPP under constant stirring, resulting in the formation of nanoparticles. The size of the polymeric nanoparticles varies with pH—Lower pH values yield smaller particles (100 nm for a pH value of 5) (Marciniak et al., 2020). Hybrid nanoparticles can be made by adding a second compound such as poly (acrylic acid) or carboxymethyl cellulose; however, this approach is only limited to chitosan-based drug carriers (Lai et al., 2014).

### 2.3 Emulsion evaporation and diffusion methods

The synthesis of nanoparticles using the evaporation-based method begins with emulsifying an organic solvent and a polymer in an aqueous phase with a stabilizer. Next, high shear stress is applied to break emulsion droplets into smaller ones, and subsequently removing the solvent through evaporation, causing polymer precipitation and nanoparticle formation. Ostwald ripening can occur in the absence of high shear stress, resulting in droplet migration and aggregation (Wik et al., 2020). In this approach, the size of the nanoparticles is dependent on the polymer concentration; for instance, concentrations of 0.79, 2.5, 5, and 7.5% yielded particles sizes of 220, 178, 177, and 236 nm, respectively (Julienne et al., 1992). Additionally, the particles size is also influenced by the organic solvent ratio; Julienne et al. have shown that solvent ratios of 10, 25, and 40% (v/v) resulted in particles size of 106, 111.2, and 130 nm, respectively.

On the other hand, diffusion-based emulsion involves the introduction of an emulsifier into the solution containing polymer and organic solvent, followed by the addition of significant amounts of water during mixing. This process leads to the formation of oil-in-water emulsions, and the polymeric nanoparticles are subsequently produced through the evaporation of the organic solvent during the mixing process (Lai et al., 2014). In this approach, several parameters control the nanoparticles’ size, including the polymer concentration, mixing speed, the stabilizer, and the temperature (Hickey et al., 2015; Mulia et al., 2019; Pulingam et al., 2022). While this approach is ideal for hydrophobic drugs as they dissolve in the solvent leading to efficient encapsulations, double emulsion is implemented in the case of hydrophilic drugs, to overcome the effect of polar solvent that lowers the encapsulation efficiency in single emulsions (Bilati et al., 2005; Lai et al., 2014).

The synthesis of polymer-based nanoparticles for drug delivery in bone cancer therapy involves various approaches and techniques. Both synthetic and natural biodegradable polymers are utilized, each offering distinct advantages. The synthesis methods discussed in this section include water-in-oil emulsification, ionotropic gelation, and emulsion evaporation/diffusion. These methods allow for the control of nanoparticle size and drug loading efficiency through the manipulation of parameters such as polymer concentration, pH, solvent ratio, and mixing speed. Each method presents unique benefits and considerations for encapsulating hydrophobic or hydrophilic drugs. By understanding and applying these synthesis strategies, researchers can tailor the properties of polymer-based nanoparticles to optimize their effectiveness in targeted drug delivery for bone cancer therapy.

## 3 Targeting mechanisms

Chemotherapy and excision have proven effective in increasing the survival rate of osteosarcoma patients, with the added benefit of preserving their limb in 75% of the cases. However, despite the demonstrated efficacy of chemotherapy (Ferrari et al., 2014; Isakoff et al., 2015), there are challenges in delivering efficient doses to ensure drug diffusion into the tumor cells. This is mainly due to two factors: 1) the physical properties of bones (such as low permeability, hardness, and low blood flow); and 2) the direct correlation between high drug concentration and toxicity, which can lead to necrosis (Li et al., 2016). Therefore, specific targeting of the osteosarcoma location is crucial in the treatment process. In general, targeting cancerous bone locations can be classified as passive or active, depending on the method used.

### 3.1 Passive targeting

In this strategy, drug molecules reach tumors (especially when their size is 1–2 mm^3^) through their structurally defective and discontinuous vasculature. Due to the tumors’ high interstitial pressure and malfunctioning lymphatics, the molecules are retained at the tumor site (Popwell et al., 2014): this phenomenon is referred to as enhanced permeability and retention (EPR) (Nafo, 2022). Thus, reducing the particles size (<50 nm) increases their accumulation at the bone tumor location (Hoshyar et al., 2016). Furthermore, modifying the particles’ surface increases their accumulation in the bone tumor sites thanks to their reduced clearance by the spleen and liver. Sou et al. have shown that modifying liposomes with PEG reduced their intake by the liver and increased their distribution in the bone marrow (Li et al., 2016). Moreover, PEGylated particles were also reported to have prolonged cycle time in the blood in osteosarcoma therapeutic applications.

### 3.2 Active targeting

Active targeting strategies are dependent on the utilization of targeting moieties, such as ligands that are affixed to the surface of nanoparticles to accomplish active targeting payloads at the location of the tumor cells. For bone targeting applications, nanoparticles are equipped with a targeting ligand that binds specifically to hydroxyapatite (HA) to transport the therapeutic drugs. In polymeric nanoparticles, pamidronate, Bisphosphonates (BP), and aptamers are commonly employed ligands (Yu et al., 2015; Rudnick-Glick et al., 2016; Yin et al., 2016; Fang et al., 2017; Yan et al., 2022). Yin et al. (2016) coated doxorubicin-loaded polyactide nanoparticles with bone-seeking pamidronate. They showed that in animal models, these nanoparticles present a remarkable buildup and prolonged retention time, resulting in significantly reduced localized osteosarcoma progression.

The targeting capability of BP is attributed to its strong affinity to Ca^2+^ions. Thus, when they decorate PEGylated nanoparticles that are used to deliver doxorubicin to Saos-2 human osteosarcoma cell xenograft mouse model, the conjugated nanoparticles demonstrate 40% greater inhibition of tumor growth (Rudnick-Glick et al., 2016).

Aptamers are short, single-stranded oligonucleotides designed for precise targeting applications via systematic evolution of ligands (Wang et al., 2018). This process allows for the discrimination and monitoring of subtle differences in molecular expression between phenotypic states of homologous cells (Zueva et al., 2011), including recognizing cancer cells from normal cells, and cancer cells of different metastatic levels (Duan et al., 2016; Jia et al., 2016). When Yu et al. (2015) conjugated CD 133 aptamers to polymeric nanoparticles for drug delivery of osteosarcoma cancer stem cells, the nanosystems showed effective targeting and inhibition to osteosarcoma growth by killing CD 133^+^ cells.

The effective treatment of osteosarcoma relies on the precise targeting of the tumor location. While chemotherapy and excision have improved survival rates and limb preservation, challenges remain in delivering efficient doses due to the unique properties of bones and the risk of toxicity. Targeting mechanisms, both passive and active, offer promising solutions to overcome these obstacles. Passive targeting takes advantage of EPR in tumors, along with particle size reduction and surface modifications to increase drug accumulation at the bone tumor site. Active targeting strategies utilize ligands affixed to nanoparticles, such as pamidronate, BP, and aptamers, to specifically bind to hydroxyapatite and transport therapeutic drugs. These active targeting approaches have shown remarkable results in reducing osteosarcoma progression and inhibiting tumor growth in animal models. Further research and development in targeting mechanisms hold great potential for improving the efficacy and precision of osteosarcoma treatment.

## 4 Drug release mechanisms

Although there are several release mechanisms associated with drug-loaded nanosystems, in osteosarcoma therapeutic applications, the controlled release mechanisms mainly revolve around two strategies: internal stimulation and external stimulation.

### 4.1 Internal stimulation strategies

The design of the nanocarriers adopting this strategy relies on the tumor microenvironment conditions to release drugs including the environment’s acidity (pH), oxidative stress, and enzymatic interactions.

The pH of tumors range is 6.5–7.2, which is lower than that of healthy tissues (7.4); this is primarily due to the tumors microenvironment’s hypoxic nature, nutrient deprivation, and accumulation of acidic metabolites (Liu et al., 2021). pH-responsive nanoaparticles achieve controlled drug delivery into tumor tissues by selectively releasing drugs in response to the lower pH conditions of the tumor environment. An example of such nanosystems are the LAPONITEs/polymer nanohybrids developed by Wang et al. (2014). These nanohybrids not only demonstrate excellent biocompatibility and physiological stability, but also exhibit sustained pH-responsive release of release of doxorubicin. In a physiological environment (pH 7.4), the nanohybrids released doxorubicin steadily, while in locations where pH was 5.0, the drug was rapidly released demonstrating higher toxicity against the CAL-72 cells compared to using free doxorubicin.

Elevated levels of hypoxia are an inherent hallmark of cancer tumors, which was reported to be associated with the increased production of reactive oxygen species (ROS) in cancer cells (100 e−06 M) (Perillo et al., 2020), higher than healthy tissues (20 e−09 M) (Trachootham et al., 2009). Therefore, drug delivery from ROS-sensitive nano-carriers enhances the controlled release of therapeutics, and increases the exposure of cancer cells to the drug molecules (Xu et al., 2017). Xu et al. (2017) created ROS-sensitive nanocomposites composed of a polyprodrug core and PEG shell, which were surface-encoded with the internalizing arginine glycine-aspartic acid (iRGD). The PEG shell enabled sustainable circulation, while iRGD promoted tissue penetration. These nanoparticles exhibited a response to intracellular ROS by triggering the release of the drug via chain breakage. This drug release mechanism led to significant inhibition of cancer cell growth in both *in-vitro* and *in-vivo* tests.

Another tumor-specific targeting strategy is using enzyme-responsive nanoparticles. In the tumor microenvironment, the expression levels of various enzymes such as metalloproteinase, heparanase, and proteases, can undergo changes that can be utilized for achieving targeted drug accumulation at tumor sites through enzyme-responsive drug release (Mura et al., 2013). Huang et al. (2019) developed an enzyme-responsive nanoparticle, namely paclitaxel (PTX)-DOTAP@alloferon-1-heparin/protamine, for the treatment of tumors expressing high levels of heparanase-1, a characteristic feature of highly metastatic osteosarcoma. The outer layer of this nanosystem was designed to disassemble upon the degradation of heparin by the extracellular matrix’s highly expressed heparanase-1, thereby releasing PTX and protamine for effective tumor therapy. *In vitro* and *in vivo* experiments confirmed the efficacy of this nanosystem in the treatment of osteosarcoma.

### 4.2 External stimulation strategies

In addition to internal stimulators, stimuli-responsive nanosystems have a demonstrated capacity for controlled drug delivery utilizing external stimulators such as magnetism, ultrasound, and light.

The effectiveness of chemotherapy in cancer patients can be enhanced by inducing local hyperthermia using magnetic nanoparticles. Although various polymeric nanoparticles have exhibited features related to drug loading and release, such as swelling and deswelling, in response to external thermal stimuli, their ability to penetrate deep into tissues and activate the nanoparticles is often limited. Conversely, the utilization of paramagnetic materials can generate the necessary levels of hyperthermia upon exposure to an alternating magnetic field (AMF), that is, capable of penetrating deep tissue, enabling the design of magnetically responsive nanocarriers with controlled drug release. Jalili et al. (2017) developed nano-gel composites composed of a poly (N-iso-propylacrylamide-co-acrylamide) (poly (NIPAM-co-AM)) shell encapsulating a core of citric acid coated iron oxide nanoparticles. The nanosystems provided controlled on-demand delivery of doxorubicin when exposed to AMF, in addition to the protection provided by the gel shell that limits the release of doxorubicin in the absence of high temperature. These characteristics make these nanosystems an ideal option for therapeutic applications of osteosarcoma.

In addition to AMF, ultrasound waves can safely and non-invasively penetrate and propagate through living tissues. Furthermore, ultrasound has been demonstrated as an effective method for triggering drug release from nanocarriers (Wilson and Kubanek, 2021). The efficacy of ultrasound in triggering drug release stems from the ability to safely focus the energy waves into specific small volumes, deep inside the body (Ibsen et al., 2013). The mechanism underlying drug release from nanocarriers involves the thermal effect induced by ultrasound. In particular, application of high-frequency ultrasound to PEGylated nanoparticles causes detachment of the PEG shell, which subsequently facilitates interaction between the nanoparticle core and cancer cells, leading to the release of the encapsulated chemotherapeutic agents. This mechanism was reported by Paris et al. (2018), who decorated positively charged and amine functionalized mesoporous silica nanoparticles (loaded with topotecan) with PEG using thermo-sensitive linkers (4,4′-azobis (4-cyanovaleric acid). Upon exposure to ultrasound, the generated heat resulted into the cleavage of the linkers, which separated the PEG chains from the nanoparticles, exposed their positively charged surface, and eventually lead to the uptake of nanoparticles and significantly increased their toxicity in human osteosarcoma cells.

Light-sensitive nanoparticles are a novel approach to drug delivery that employs light as an external stimulus to regulate drug loading and release. Compared to other stimuli, light is considered a gentle, non-invasive, and highly precise external trigger that minimizes toxicity and reduces side effects (Beauté et al., 2019; Rapp and DeForest, 2021). In this regard, Chen et al. (2021) developed a light-responsive nano-micelle drug delivery system (Poly-Dox-M). The nano-micelles structure contains PEG, which acts as a polymer shield in the bloodstream, ensuring a stable nanostructure and enhancing tumor targeting while reducing the systemic toxic effects of doxorubicin. The drug-loaded micelles accumulate at the tumor site through the EPR effect. When exposed to light irradiation, the micelles experience a rapid structural change at the tumor site, shedding the PEG shell, and releasing the drug, which enhances doxorubicin uptake by the tumor cells, thereby increasing the chemotherapy’s efficacy. The study outputs demonstrate that Poly-DOX-M exhibits sensitive light response characteristics, excellent anti-cancer effect, preferable biosafety, and favorable cellular uptake.

The development of effective drug release mechanisms is crucial for optimizing osteosarcoma therapeutic applications. Internal stimulation strategies, such as pH-responsive, ROS-sensitive, and enzyme-responsive systems, take advantage of the tumor microenvironment to achieve controlled drug release. These approaches allow for selective drug delivery and increased exposure of cancer cells to therapeutic agents, leading to enhanced efficacy. External stimulation strategies, including magnetic field, ultrasound, and light, provide additional means to regulate drug release from nanosystems. By utilizing external stimuli, such as AMFs, ultrasound waves, and light irradiation, controlled drug release can be achieved with precision and minimal invasiveness. These external stimulation strategies offer opportunities for targeted drug delivery and increased treatment efficacy. Overall, the development of diverse and innovative drug release mechanisms holds great promise for advancing osteosarcoma treatment and improving patient outcomes. Further research and exploration in this field are necessary to harness the full potential of these strategies in clinical settings.

**FIGURE 1:**
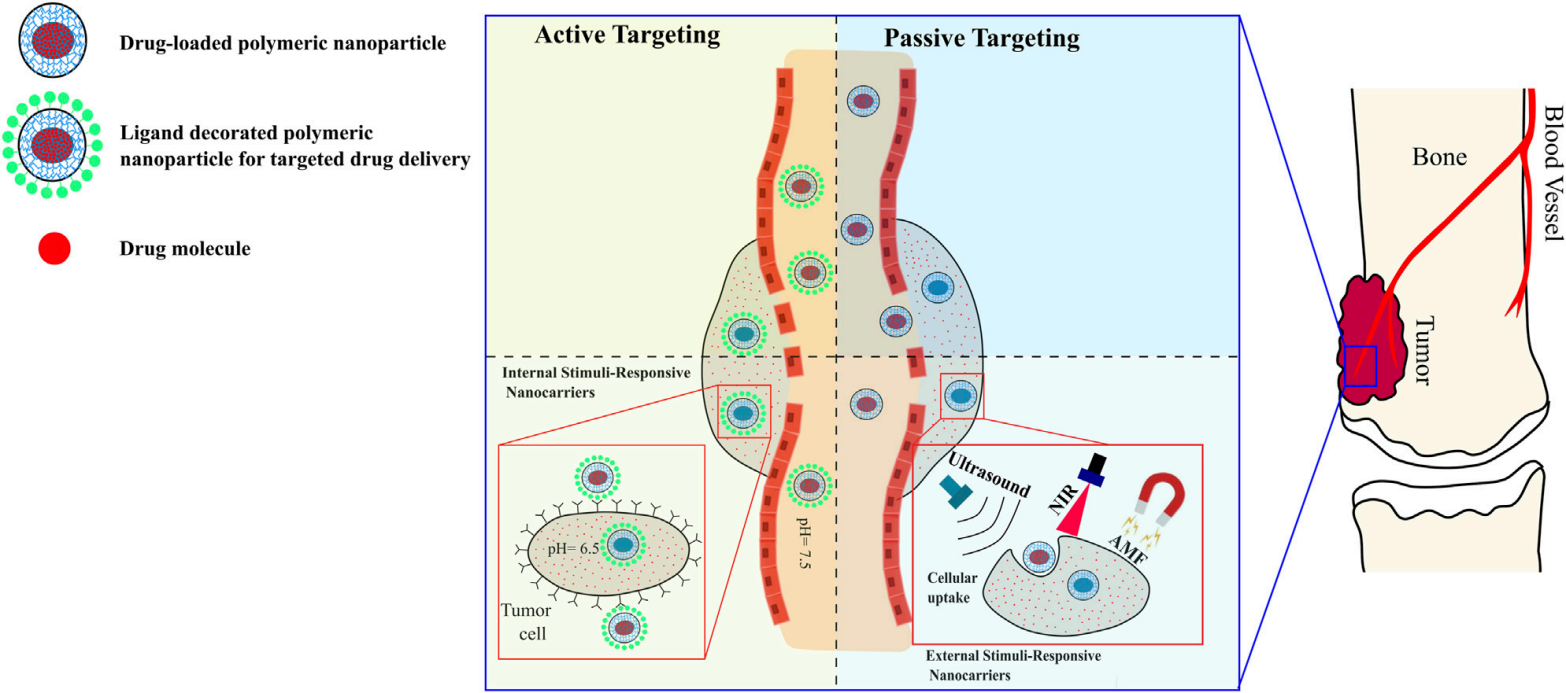
Schematic illustrations of targeting mechanisms of polymer-based nanocarriers against osteosarcoma, and examples of drug release strategies. Processing nanoparticles to be internal-responsive or external-responsive is applicable for passive and active targeting.

Figure 1 shows a schematic illustration of active and passive targeting with different drug release mechanisms.

## 5 Biodistribution, clearance, and nanotoxicity

The distribution of nanoparticles in the body is a challenge in targeted drug delivery via nanocarriers, as it can accumulate in unintended areas or target different sites. This scenario is influenced by barrier properties and the formation of a protein corona around the nanoparticles when they come into contact with biological bodies (Riviere, 2013). Therefore, to achieve efficient transport and biodistribution of nanoparticles, researchers have used receptor-targeting ligands, peptides, and cell-mediated drug delivery to control the transport of therapeutic nanoparticles. Other approaches include using stimuli-responsive polymeric nanoparticles, surface-modified nanoparticles, and improved imaging modalities for monitoring nanoparticles distribution (Barua and Mitragotri, 2014; Das et al., 2020).

Another challenge encountered by polymeric nanoparticles to deliver the chemotherapeutics to sites of osteosarcoma is clearance—A defense mechanism in which specific tissues in the liver, spleen, and kidneys (the reticuloendothelial system) systematically clear particles and soluble substances from blood circulation. Research has shown that coating/decorating the nanoparticles with PEG minimizes clearance and extends their half-life during circulation (Suk et al., 2016).

In the realm of nanotechnology-based drug delivery for osteosarcoma therapy, designing nanoparticles that evade clearance by the immune system is a critical challenge. However, even once this challenge is addressed, researchers must consider the physicochemical properties of the nanoparticles, such as particle size, surface charge, hydrophobicity, drug binding, encapsulation rate, and bioavailability, since these properties are associated with varying levels of cytotoxicity (Maleki et al., 2021; Wu et al., 2022). To address these challenges, hybrid nanoparticles, such as peptide-decorated polymeric micelles, are being developed. This strategy holds promise in overcoming conventional challenges, such as burst and premature drug release, limited drug loading capabilities, and toxicity resulting from inorganic cores and chemotherapeutic molecules (e.g., doxorubicin). For instance, Fang et al. (2017) synthesized micelles using biodegradable polymers (size 46–73 nm) that were functionalized with RGD peptides. The resulting nanoparticles exhibited improved cellular targeting specificity towards osteosarcoma cells. Furthermore, these polymeric micelles showed drug loading and release efficiencies of 73% and 63%, respectively, over a period of 60 h under physiological conditions. Overall, drug-loaded polymeric micelles hold great potential as a nanoplatform for targeted chemotherapeutics in the treatment of osteosarcoma.

Biodistribution, clearance, and nanotoxicity are critical factors to consider in the development of nanocarriers for targeted drug delivery in osteosarcoma therapy. Achieving efficient transport and biodistribution of nanoparticles requires the use of receptor-targeting ligands, peptides, and cell-mediated drug delivery. Strategies such as surface modification, stimuli-responsive polymers, and improved imaging techniques have been employed to enhance nanoparticle distribution and overcome potential off-target effects. Clearance by the reticuloendothelial system poses a challenge to nanocarriers, but coating nanoparticles with PEG has been shown to minimize clearance and prolong circulation time. Nanoparticle cytotoxicity is influenced by various physicochemical properties, necessitating careful consideration during nanoparticle design. Hybrid nanoparticles, such as peptide-decorated polymeric micelles, offer a promising approach to address these challenges by providing improved cellular targeting specificity, controlled drug loading, and release capabilities. The development of drug-loaded polymeric micelles as a nanoplatform holds significant potential for targeted chemotherapeutics in osteosarcoma treatment, offering hope for improved therapeutic outcomes and reduced toxicity.

## 6 Discussion and future directions

The quest to synthesize polymeric nanoparticles for the treatment of osteosarcoma has been the subject of numerous studies in the past few years. However, the methods employed in producing these particles often yield inconsistent and discordant results in terms of particle size (Li et al., 1998; Kaldybekov et al., 2019; Sarma et al., 2021). Factors such as polymer type, temperature, homogenization process, drug loading efficiency, and emulsion conditions all contribute to the variation in the size of the nanoparticles. Particles with diameters less than 200 nm are less likely to be cleared, and it is essential to produce particles of uniform size for their use in preclinical and clinical trials (Lai et al., 2014). Moreover, many synthesis procedures in the literature follow formulae that contain toxic reagents; for instance, the synthesis of PLGA nanoparticles require the use of acetone (Wu et al., 2022). Similarly, ethanol is an integral agent in the synthesis of polydopamine nanoparticles (Amin et al., 2018), in addition to several other chemicals that are not easily removed through repeated washing and rinsing (Wu et al., 2022). Although toxicity evaluations and animal models have been used to assess the safety of polymeric nanoparticles, only a few have been approved by the US Food and Drug Administration for clinical trials (Piscatelli et al., 2021). To address these issues, research must focus on three main domains hindering the transition of polymeric nanocarriers to clinical practice: 1) the long-term biological safety and toxicity, 2) the elevated rate of immune clearance, and 3) the inadequacy of dependable experimental findings derived from animal models and clinical patients. Future studies must overcome these challenges to facilitate the secure and efficient utilization of polymeric nanoparticles for the management of osteosarcoma.
